# Electrodeposited Reduced Graphene Oxide Enables Long‐Term Memory in Neuromorphic Ambipolar Electrolyte‐Gated Transistors

**DOI:** 10.1002/smll.202502768

**Published:** 2025-05-16

**Authors:** Maryam Abouali, Federico Rondelli, Matteo Genitoni, Mauro Murgia, Michele Di Lauro, Luciano Fadiga, Fabio Biscarini

**Affiliations:** ^1^ Center for Translational Neurophysiology of Speech and Communication Fondazione Istituto Italiano di Tecnologia (IIT‐CTNSC) via Fossato di Mortara 17/19 Ferrara 44121 Italy; ^2^ Sezione di Fisiologia Dipartimento di Neuroscienze e Riabilitazione Università di Ferrara via Fossato di Mortara 17/19 Ferrara 44121 Italy; ^3^ National Research Council Istituto per lo Studio dei Materiali Nanostrutturati (CNR‐ISMN) via Gobetti 101 Bologna 40129 Italy; ^4^ Dipartimento di Scienze della Vita Università di Modena e Reggio Emilia Via Campi 103 Modena 41125 Italy

**Keywords:** long‐term plasticity, neuromorphic organic devices, nonvolatile memory, reduced graphene oxide

## Abstract

Ambipolar transistors, capable of conducting both electrons and holes, enable the simplification of circuit design by reducing the number of constituting units in circuits and opening new possibilities for low‐power electronics, reconfigurable logic circuits, and memory devices. 2D ambipolar semiconductors as graphene and its derivatives, are particularly advantageous in bioelectronics, for their high sensitivity in label‐free sensors and their biocompatibility. Here, a novel method for fabricating electrolyte‐gated transistors based on reduced graphene oxide (rGO‐EGTs) is proposed, which enables precise control over the thickness of deposited rGO. Such rGO‐EGTs act as a neuromorphic unit that exhibits tailorable long‐term plasticity when driven with pulsed voltage. By applying different numbers of voltage pulses and acting on their amplitudes, it is possible to program multilevel memory with retention timescales over tens of minutes and 6.60 µS writing resolution. This long‐term plasticity makes our rGO‐EGT promising for nonvolatile memory, computing, and plasticity‐based signal pattern recognition.

## Graphical abstract

An electrolyte‐gated transistor based on reduced graphene oxide, which is prepared via a facile and straightforward way, enables ambipolar conduction with a near‐zero charge neutrality point. Upon administration of various pulse experiments, the device is capable of performing outstanding neuromorphic functions such as long‐term plasticity and multistate memory.

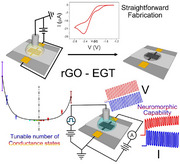

## 1 Introduction

Organic transistors typically rely on unipolar organic semiconductors, either p‐type – conducting holes – or n‐type – conducting electrons. Ambipolar transistors – conducting both electrons and holes depending on the gating conditions – enable circuit design simplification, leading to space‐ and power‐efficiency in circuitry by reducing the number of components. Examples of this approach have been reported in the cases of ambipolar light‐emitting transistors^[^ ^1^ ^]^ and ambipolar flash memory devices.^[^ ^2^ ^]^ Ambipolarity, jointly with charge carriers’ (de‐)trapping kinetics, can be exploited to mimic bidirectional signal transmission as well as neurotransmitter intake/release in biological synapses.^[^ ^3^, ^4^ ^]^ This makes them promising candidates for developing neuromorphic computing systems that emulate the logic functionality of the brain circuitry.

Various semiconductors have been explored for ambipolar devices: conjugated polymers,^[^ ^5^ ^]^ organic molecules,^[^ ^6^ ^]^ inorganic‐organic hybrid materials,^[^ ^7^ ^]^ and 2D materials.^[^ ^8^ ^]^ Among the latter, graphene and its derivatives could be used as both active moieties and electrode materials.^[^ ^9^ ^]^ This work focuses on reduced graphene oxide (rGO), which features improved scalability and ease of functionalization with respect to graphene, thereby imparting enhanced application versatility.^[^ ^10^, ^11^, ^12^ ^]^ The typical route to engineer rGO‐based transistor channels foresees surface treatments, drop‐casting of graphene oxide, and subsequent reduction to rGO. This complicates the fabrication process and often yields incompletely reduced channels. Here, we propose a novel, straightforward method for fabricating rGO‐based electrolyte‐gated transistors (rGO‐EGTs), via electrochemical reduction from GO dispersions using surface‐patterned interdigitated electrodes. This results in the spontaneous formation of a rGO thin film, bridging the source and drain contacts. Our method enables thickness control and tailoring of the charge neutrality point (*V_CNP_*). *V_CNP_* is a key descriptor of graphene‐based transistors, indicating the condition in which p‐ and n‐transport are equal.^[^ ^13^ ^]^ The proposed rGO‐EGTs exhibit stable ambipolar response and transconductances > 300 µS for both conductance branches. When used as neuromorphic units, rGO‐EGTs show long‐term plasticity (LTP), with time constants and magnitudes strongly depending on the doping level and, hence, they can be tailored by acting on the gate DC bias.^[^ ^14^ ^]^ The proposed rGO‐EGTs strongly respond to ion currents with transient transduction and long‐term integration of input signals, acting as nonvolatile multistate memories, paving the way toward in situ real‐time signal/information processing/storage in bioelectronic applications.

## 2 Results and Discussion

### 2.1 Direct Electrodeposition of rGO for EGTs

**Figure 1:**
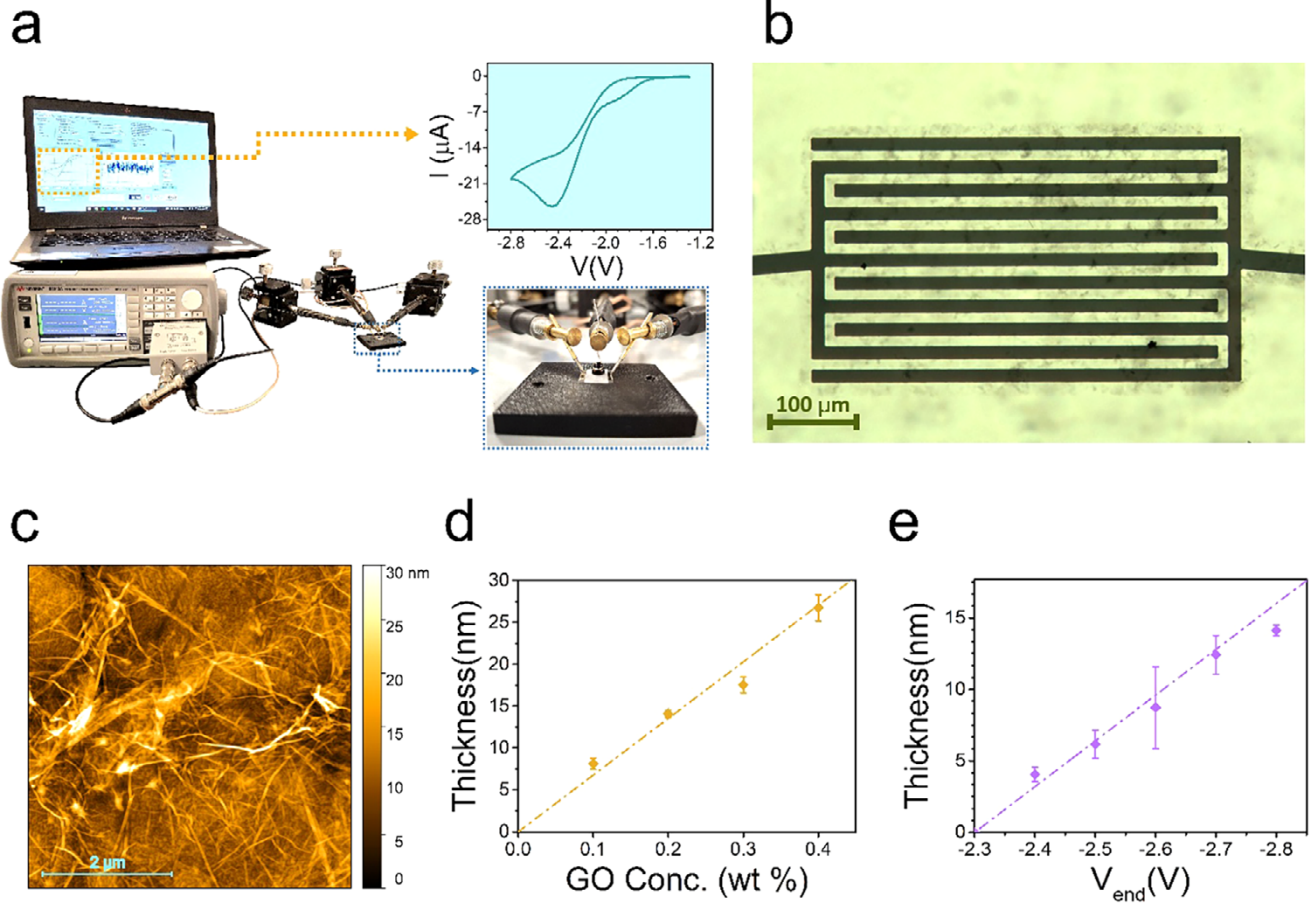
Fabrication setup and morphological characterization of rGO‐EGTs. a) electrodeposition setup for in situ fabrication of rGO–EGT, with insets showing the cyclic voltammogram of GO electrochemical reduction (top) and a magnification of the test pattern connection (bottom). b) Optical micrograph of the interdigitated electrodes after rGO channel electrodeposition. c) AFM image of rGO channel morphology. d,e) Dependency of the thickness of electrodeposited rGO channels (d) on the concentration of GO dispersions (n_sample_ = 3, standard error of the mean as error bars) on *V_end_* (n_sample_ = 3, standard error of the mean as error bars). Dash‐dotted lines are linear fitting, used as guides for the eye.

Electrodeposition of rGO channels on the interdigitated surface‐patterned gold electrodes was performed using single‐run cyclic voltammetry (CV) starting from graphene oxide (GO) dispersion (0.2 wt.% in water, see Experimental Section for details). The electrodeposition setup features short‐circuited surface electrodes as the working electrode (WE) and a Pt‐wire as a self‐referenced counter electrode (CE/RE) (**Figure** **1**a). The irreversible reduction of GO to rGO occurs at ≈−2.45 V vs CE /RE (Figure 1a, inset), leading to the growth of rGO on the electrodes’ surface. A conductive rGO‐channel is formed between the interdigitated contacts (Figure 1b), with an average resistance equal to 2.3 ± 1.2 kΩ (n*_samples_* = 10).

The topography of such rGO films was assessed with Atomic Force Microscopy (AFM). Figure 1c shows a multilayer‐flake structure, with thicker edges and higher ripples superimposed on flat regions. Average roughness equals 3.1 ± 0.2 nm (n*_samples_* = 3).

The proposed procedure is sought to enable quantitative control over the thickness of electrodeposited rGO by adjustment of operational parameters, such as the number of electrodeposition cycles, GO concentration, and selected electrochemical procedure. In this work, we focused on single‐run CV, investigating the dependency of rGO thickness on GO concentration and on the electrodeposition voltage range.

To estimate thickness, rGO films were mechanically scratched, and AFM was performed across the scratch edge (Figure S1, Supporting Information).

The thickness of films obtained from varying GO dispersion concentrations is reported in Figure 1d, unveiling a linear relationship in the investigated range, [0.1 wt.%: 0.4 wt.%]. Performance comparison between rGO‐EGTs built on these films (Figure S2, Supporting Information) shows that 0.2 wt.% GO dispersions yield optimal steady‐state transfer characteristics in terms of ambipolarity (i.e., symmetry), ON/OFF ratio, and *V_CNP_* (Table S1, Supporting Information). Aiming at finer thickness tuning, we investigated the effect of the CV range for this concentration. We kept the positive boundary of the scan fixed at −1.3 V vs CE/RE and varied the negative boundary, *V_end_*, between −2.0 and −2.8 V vs CE/RE. The corresponding thickness vs *V_end_* trend is presented in Figure 1e, exhibiting a linear dependency. No electrodeposition can be observed for |*V_end_* | < −2.4 V (i.e., thickness = 0 nm, for −2.3 V ≤ *V_end_* ≤ −2.0 V). Also, the exchanged charge during electrodeposition, Q, estimated by CV integration over time, follows an increasing trend versus *V_end_* (Figure S3, Supporting Information).

The envisioned neuromorphic rGO‐EGT operations require coupling optimal ambipolar response in the steady state – achieved with 0.2 wt.% GO dispersions – with a sufficient amount of ion intercalation/adsorption sites (i.e., the highest possible thickness at the selected GO concentration, achieved for *V_end_* = −2.8 V). Steady‐state and neuromorphic behavior of these devices is characterized in the upcoming sections.

### 2.2 Electrical Characterization of Proposed rGO‐EGTs

**Figure 2:**
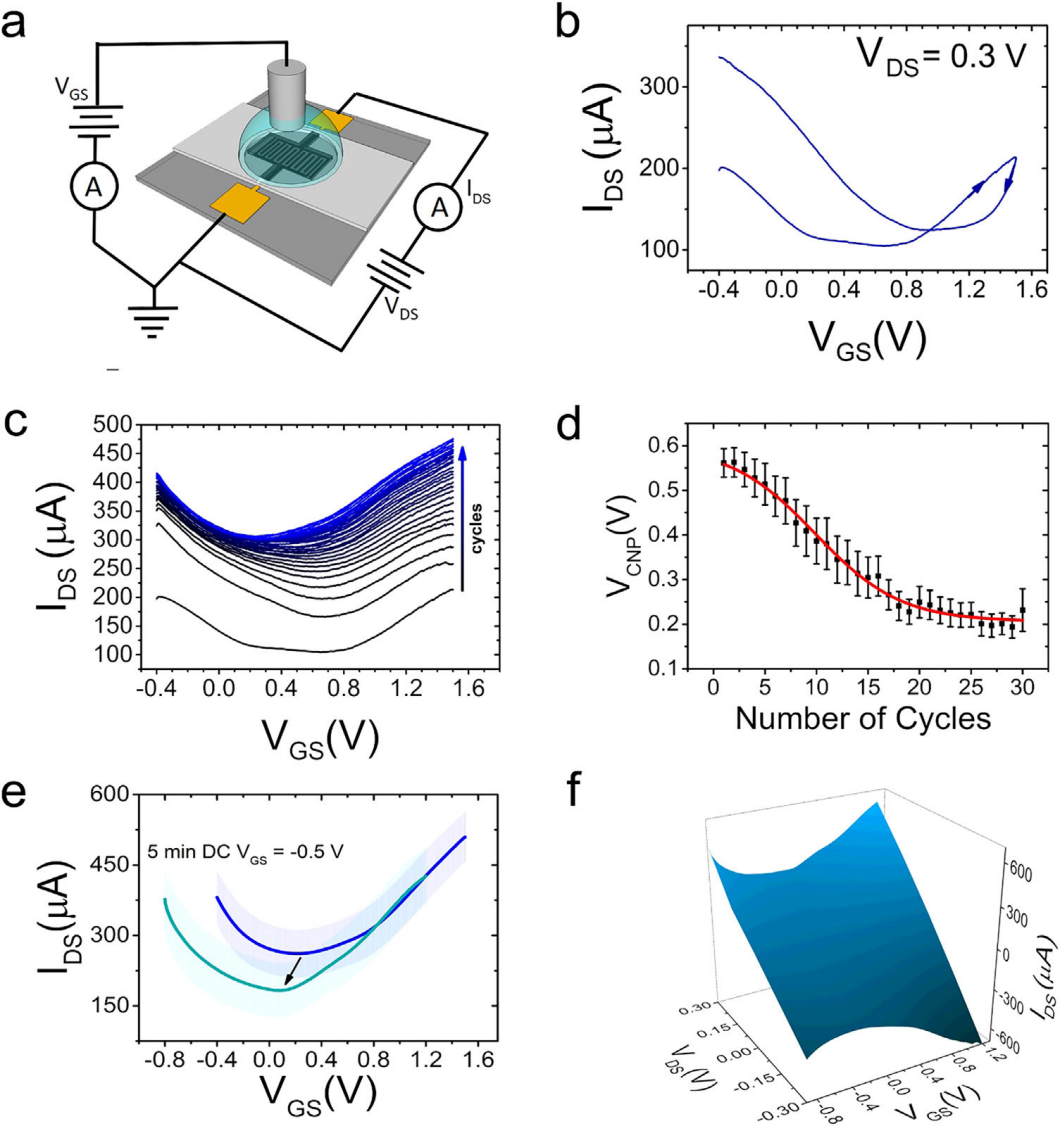
*I–V* characteristics of rGO‐EGTs. a) rGO‐EGT schematics for steady‐state device characterization. b) Transfer characteristic of proposed rGO‐EGT. c) Evolution of transfer curves upon iterative *V_GS_* cycling (starting from black solid line to blue solid line). d) Dependency of *V_CNP_* vs the number of gate sweep cycles (n_sample_ = 8, standard error of the mean as error bars). e) Transfer characteristics of rGO‐EGT before (blue solid line) and after the DC gate voltage reconditioning step (emerald green solid line) (n_sample_ = 8, standard error of the mean as error bars). f) 3D representation of *I–V* characteristics of a representative rGO‐EGT.

rGO‐EGTs were characterized in common‐source/common‐ground configuration (**Figure** **2**a), using the rGO‐bridged electrodes as source and drain contacts, and a Pt‐wire as gate electrode bathing in a 1M PBS gating electrolyte. Figure 2b is the representative transfer characteristic of a freshly deposited rGO‐EGT. It is possible to observe the hallmarks of ion‐gated ambipolar charge transport in the channel: positive gate voltages cause the accumulation/intercalation of cations close to/inside the rGO film, resulting in increased electron transport, i.e. n‐type branch; the opposite occurs for negative voltages, which cause cation depletion/anion accumulation, resulting in increased hole transport, i.e. p‐type branch. This behavior occurs already upon the first gate‐biasing cycle, albeit with a large region (at positive *V_GS_*) in which *I_DS_* vs *V_GS_* is substantially flat. *V_CNP_*, which here can be extracted as the *V_GS_* value at minimum *I_DS_*, lies ≈0.7 V, hinting at a strong parasitic p‐doping of the channel.^[^ ^15^, ^16^ ^]^ The hysteresis behavior is peculiar, shifting between clockwise hysteresis (i.e., easier de‐doping than doping) at strongly positive *V_GS_* and counterclockwise hysteresis (i.e., easier doping than de‐doping), typical of organic semi‐conductors in electrolytes, which suggests the existence of a critical positive *V_GS_* (≈0.95 V, Figure 2b), marking the onset of a region dominated by electrostatic repulsion between incoming and already intercalated cations.

Upon repetitive cycling, intercalation/accumulation of cations in the rGO film increases electronic current, corresponding to an increased number of mobile negative charges (i.e., additional n‐doping, testified by a *V_CNP_* negative shift), and to the reduction of junction resistances between adjacent rGO sheets.^[^ ^17^, ^18^ ^]^ Typical evolution of the device response upon this iterative conditioning is shown in Figure 2c, while Figure 2d shows the corresponding *V_CNP_* vs cycle trend, demonstrating the attainment of stable device response after ≈ 20 cycles, with a *V_CNP_* negative shift greater than 300 mV. To surely achieve stability, all the devices presented herein underwent thirty conditioning cycles.

The sigmoidal trend (Figure 2d) hints at a two‐state transition between states at different free energies. Such bistable behavior may be due to the metastability of the initial state, as discussed below.

The cycle‐wise increase of the electron current, which is not quantitatively paralleled by the hole current, results in an increase of *I_DS_* at *V_CNP_* – termed OFF current – causing the ON/OFF ratio to decrease.

We address this shortcoming by designing a reconditioning procedure, involving the application of a constant negative DC bias at the gate (*V_GS_* = −0.5V, 5 min). The reconditioning procedure removes intercalated cations, yielding a significant OFF‐current lowering (i.e., ON/OFF increase). Noteworthy, reconditioning does not restore the parasitic p‐doping, instead it brings *V_CNP_* closer to 0 V, at the center of the ideal range for physiological applications. Figure 2e highlights the effects of reconditioning, showing a before‐and‐after transfer overlay. The corresponding transconductance vs *V_GS_* profiles are shown in Figure S4 (Supporting Information). Quantitative figures of merit of the final rGO‐EGT layout according to the unified analytical model of Electrolyte‐Gated Organic Transistor are reported in Figure S5 (Supporting Information), together with their evolution upon conditioning.

The final rGO‐EGTs show symmetrical ambipolar behavior over a wide range of *V_GS_* and *V_DS_* values (Figure 2f). Dependency on *V_DS_* shows a linear trend with no saturation regime and no substantial contact resistance. A significant symmetry can be observed when reversing the sign of *V_DS_*, as expected for graphene‐based transistors.^[^ ^19^, ^20^, ^21^ ^]^

We infer that the necessity of cycling and reconditioning arises from our deposition procedure, which favors kinetically‐controlled local energy minima when it comes to film structure.^[^ ^22^ ^]^ In particular, partially‐reduced GO domains embodied in the rGO film would act as *p*‐type dopants (i.e., trap‐states for *n*‐type carriers), shifting *V_CNP_* to strongly positive values as elsewhere reported.^[^ ^23^ ^]^ Upon quasi‐static cycling followed by DC reconditioning, the system is given the time and the energy to explore a broader free‐energy landscape, eventually filling trap‐states and ending up in its absolute minimum. No relevant morphological changes are observed in AFM topographies collected during cycling/reconditioning (Figure S6, Supporting Information).

### 2.3 Neuromorphic Behavior of rGO‐EGTs

**Figure 3:**
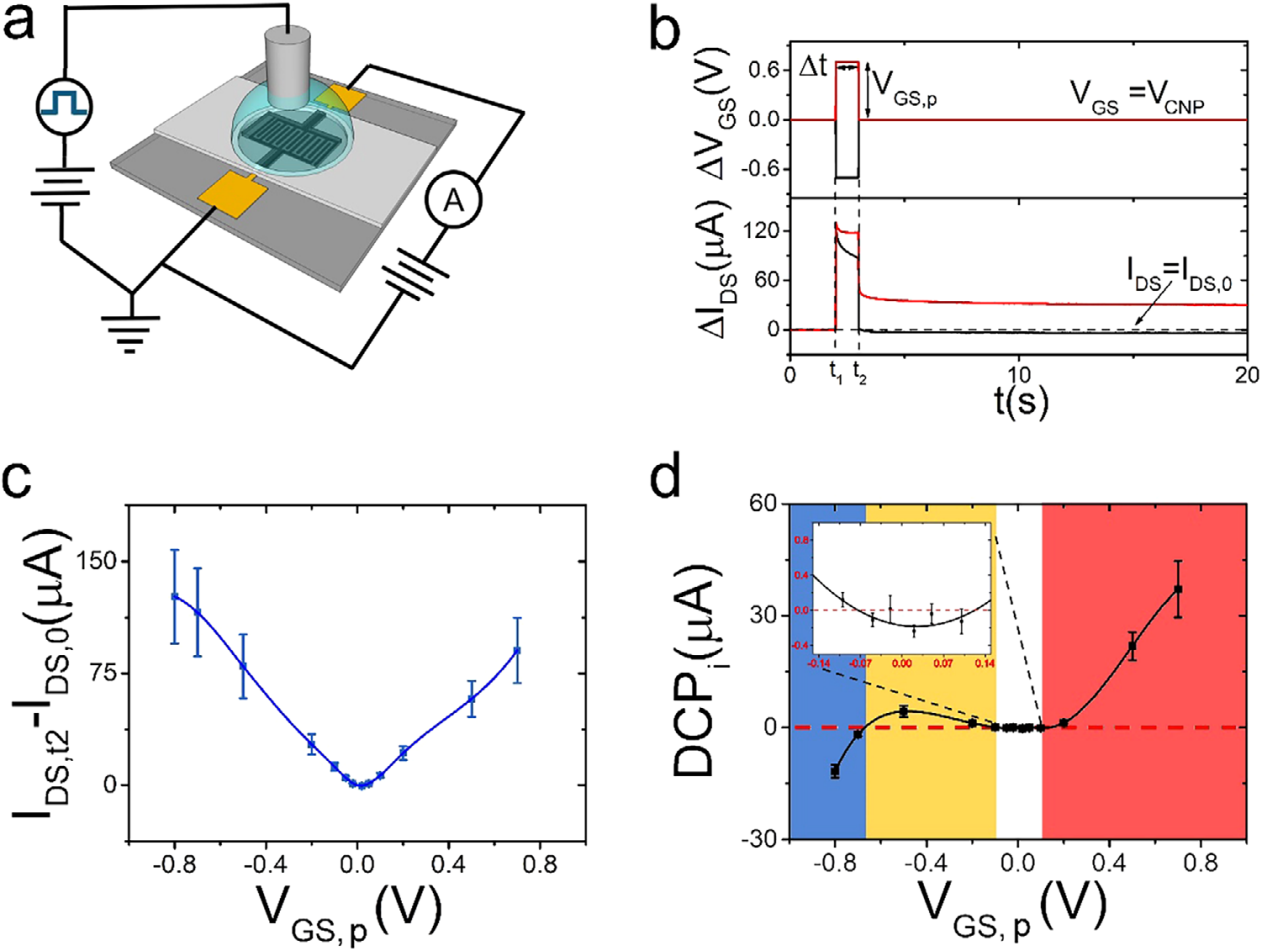
Single pulse response of rGO‐EGTs. a) rGO‐EGT schematics for neuromorphic device characterization. b) Exemplary chronovoltammogram (top) and chronoamperogram (bottom) for positive (red solid line) and negative (black solid line) single voltage pulse administration. c) Dependency of the pulse current amplitude *I_DS_* on *V_GS,p_* (n_sample_ = 4, standard error of the mean as error bars). d) *DCP_i_* vs *V_GS,p_* trend, highlighting operational windows to achieve tailorable plasticity. It is possible to distinguish four regions: i) *V_GS,p_* > 0.15V (red solid background) elicits potentiation; ii) small values of *V_GS,p_* (either positive or negative) do not elicit plasticity, with *DCP_i_* values oscillating ≈0 µA (white solid background, zoom in the inset); iii) −0.65V < *V_GS,p_* < −0.15V elicits potentiation (yellow solid background); iv) *V_GS,p_* < −0.65V elicits depression (blue solid background) (n_sample_ 4, standard error of the mean as error bars).

To explore the neuromorphic response of rGO‐EGTs, they were driven with *V_GS_* square‐pulses keeping a constant *V_DS_* = 0.3 V, while recording *I_DS_* ^[^ ^14^, ^24^ ^]^ (**Figure** **3**a). Upon such operations, rGO‐EGTs can act as neuromorphic organic devices.^[^ ^25^, ^26^, ^27^ ^]^ In analogy with biological neurons and synapses, *V_GS_* represents a presynaptic signal while *I_DS_* represents the postsynaptic signal. The transient phenomena arising in *I_DS_* upon time/frequency variations of *V_GS_* are commonly referred to as “plasticity.” The retention timescale of *I_DS_* variations after stimulus removal enables classification of neuromorphic responses between short‐term plasticity (STP) and long‐term plasticity (LTP), the former being up to tens of seconds, the latter of tens of minutes at least.^[^ ^28^, ^29^ ^]^

Keeping baseline voltage equal to *V_CNP_*, we performed a systematic evaluation of the dependency of postsynaptic current upon different amplitudes of single presynaptic voltage pulses of amplitude *V_GS,p_* (Figure 3b). Pulse duration, *Δt* = *t_2_‐t_1_*, is kept fixed at 1s.

The capacitive current spike upon positive pulse application (red solid line in Figure 3b, bottom panel) relaxes to a plateau during Δt with a trend close to a perfect exponential decay (time‐constant τ = 0.10s, stretching exponent β = 0.83, Figure S7, Supporting Information).

Figure 3c reports the difference between *I_DS_* recorded immediately prior to pulse removal, *I_DS_* (*t_2_*), and the baseline current, *I_DS_* (0), as a function of *V_GS,p_*. Due to rGO ambipolarity, *I_DS_* (*t_2_*) – *I_DS_* (*0*), is always positive and its trend mirrors the transfer characteristics of rGO‐EGTs (Figure 2e) since it arises from devices’ potentiometric sensitivity, a key parameter for the design of transconductance‐based (bio‐)sensors.^[^ ^30^, ^31^, ^32^, ^33^, ^34^, ^35^, ^36^, ^37^, ^38^, ^39^ ^]^

To quantitatively describe *I_DS_* plasticity, we introduce the Duty‐Cycle Plasticity index, *DCP_i_*, calculated according to Equation (1):

(1) $$DCPi=I_{\mathrm{DS}}\left(t_{2}+\Delta t\right)-I_{\mathrm{DS}}\left(0\right)$$

At positive *V_DS_*, *I_DS_* is always positive, and *DCP_i_* < 0 corresponds to synaptic depression, while *DCP_i_* > 0 identifies synaptic potentiation.

According to the *DCP_i_* dependency on *V_GS, p_*. (Figure 3d), positive gate pulses (ranging from + 0.2 to + 0.7 V, red background) result in *I_DS_* potentiation. Such an effect is consistent with the cation intercalation/*n*‐doping discussed above and elsewhere.^[^ ^17^, ^18^ ^]^

Conversely, strongly negative *V_GS,p_* values (−0.8 V < *V_GS,p_* ←0.65 V, blue background) give rise to negative *DCPi*)i.e., de‐doping/depression), with an overall decrease of channel conductance upon complete cation unloading, despite the potentiating effect which should be observed for anion loading.

Interestingly, for single pulses, it is possible to achieve potentiation not only for *V_GS,p_ > 0.15 V*, but also for negative *V_GS,p_* in the range of −0.65–−0.15 V (yellow background), hinting that in this region the p‐type doping of anion is dominant with respect to the n‐de‐doping. For −0.15 V < *V_GS,p_* < + 0.15 V, *DCP_i_* is close to zero (white background), identifying a region in which only STP might appear, by driving the system with stimuli at high frequency (i.e., ∆t < τ).

To understand time‐evolution of plasticities (i.e., of the ionic gradients determining channel conductance in time) we could refer to the two in‐series capacitances gating rGO‐EGTs:^[^ ^40^ ^]^ a chemical capacitance^[^ ^41^ ^]^ (dominant at positive *V_GS,p_*, when ion rearrangement involves the bulk of the channel through inter‐plane cation intercalation, with faster relaxation time), and the interfacial capacitance (dominant at negative *V_GS,p,_* when ion rearrangement regards mainly anion accumulation near rGO, with slower and more heterogeneous relaxation kinetics,^[^ ^42^, ^43^ ^]^ τ ≈ 0.87s, β = 0.43, Figure S7, Supporting Information).

To investigate the dependency of the plasticity on conditioning (as from Figure 2c) and on reconditioning (as from Figure 2e), single pulse experiments were also performed at different fabrication stages, with selected *V_GS,p_* = ± 0.7 V (Figure S8, Supporting Information). Up to 10 transfer cycles, *DCPi* increases with cycle numbers for positive *V_GS,p_*, while it decreases in the case of negative *V_GS,p_*, up to a saturation *DCPi* value, which slightly increases in magnitude after reconditioning.

**Figure 4:**
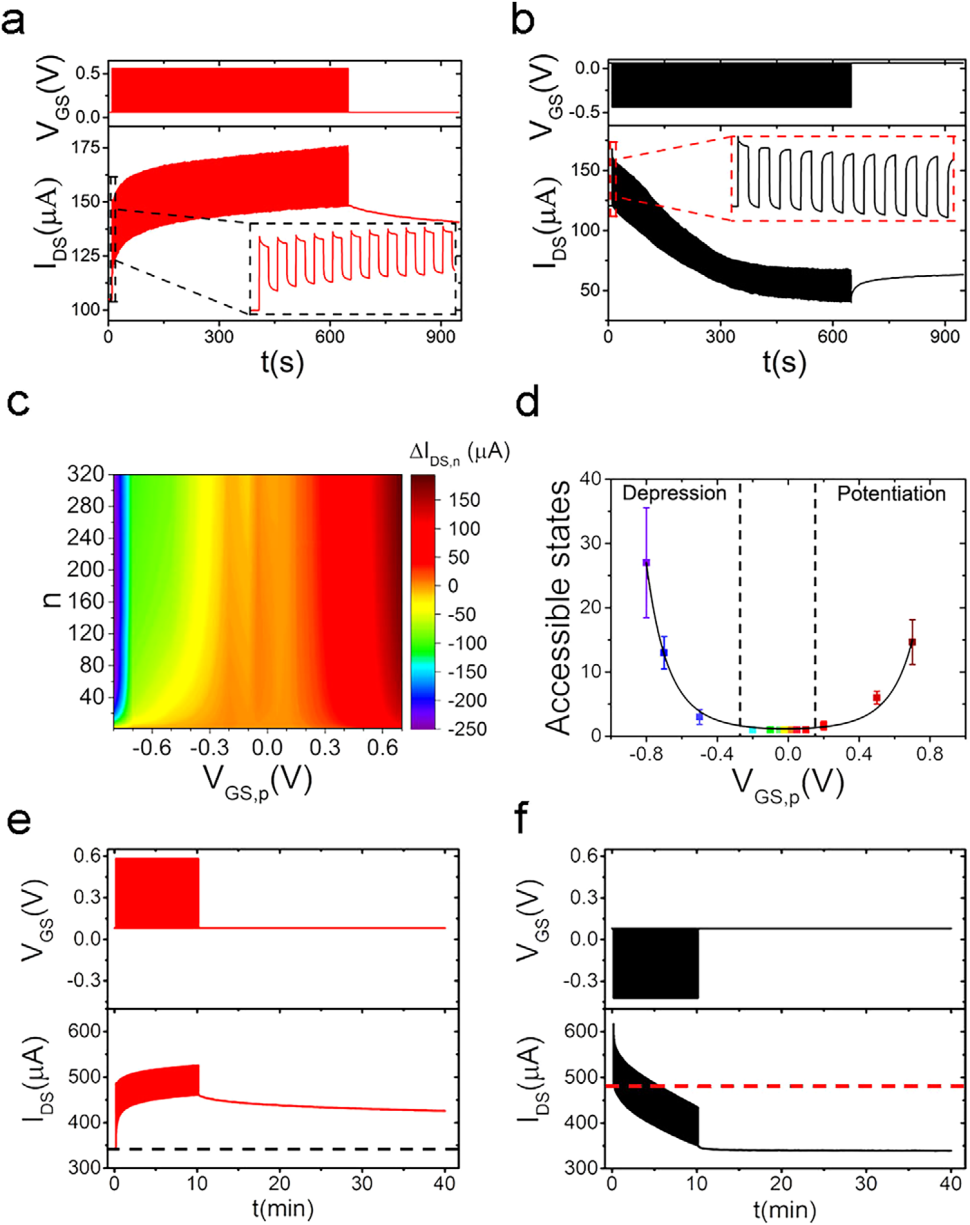
Pulse train response of rGO‐EGTs. a) Exemplary chronovoltammogram (top) and chronoamperogram (bottom) for positive train pulse administrations. b) Exemplary chronovoltammogram (top) and chronoamperogram (bottom) for negative train pulse administration. c) Dependency of the baseline current modulation (∆*I_DS,n_*) upon the number of administered pulses (*n*) and upon *V_GS,p_*. d) Number of accessible memory states as a function of *V_GS,p_* (n_sample_ = 3, standard error of the mean as error bar). The exponential of a 2nd‐order polynomial fit (black solid line) is intended as a guide for the eye. e) Positive pulse train administration (top panel, *V_GS,p_* = + 0.5 V) and corresponding *I_DS_* (bottom panel), highlighting long‐term potentiation (black dash line indicates the baseline current). f) Negative pulse train administration (top panel, *V_GS,p_* = −0.5 V) and corresponding *I_DS_* (bottom panel), highlighting long‐term depression (red dash line indicates the baseline current).

The response of rGO‐EGT was also investigated with trains of 320 pulses, keeping the baseline voltage equal to *V_CNP_* (**Figure** **4**a,b).

The contour map in Figure 4c represents the effect of *V_GS,p_* and of the number of pulses, *n*, on the potentiating/depressive plasticity effect, expressed as *∆I_DS,n_*, defined as the difference between the *I_DS_* value prior to administration of the (n + 1)th spike and the *I_DS_* value before train administration. According to this contour map, both higher *n* values and higher |*V_GS,p_* ***|*** result in a more intense plasticity, either depressive or potentiating. It is worth noticing that, at a fixed *V_GS,p_*, it is possible to observe a transition between potentiation to depression upon increasing pulse number. This is due to the loss, upon repetitive pulsing, of the dominance of p‐type doping with respect to n‐type de‐doping, already discussed as the origin of the potentiation at negative *V_GS,p_* in Figure 3d. A 2D‐representation of *∆I_DS,n_* as a function of *n* at different *V_GS,p_* values, is shown in Figure S9 (Supporting Information).

Pulse train plasticity was also assessed at different fabrication stages to unveil its dependency on conditioning/reconditioning steps. A marked *∆I_DS_* decay is observed for negative pulses, while a milder plasticity can be observed for positive pulses (Figure S10, Supporting Information). This suggests that the potentiation induced by cation loading is less influenced by channel conductivity with respect to the depression induced by cation depletion, or even by anion loading, which strongly depends on the channel's pretrain conductivity.

When aiming at rGO‐EGT‐based programmable multistate memories, the number of distinguishable/accessible conductance states shall be determined. An accessible conductance state is defined as the conductance value established after a programming pulse, if it is detectably different from the conductance value prior to the pulse, at a certain pulse frequency. To define this detectability threshold, we chose to express the number of accessible conductance states in relation to the noise of the system. Such noise has been determined by averaging the rms fluctuations of fifty‐two 150s‐long baseline traces, yielding an average noise value of 0.85 ± 0.15 µA. The noise‐limited current resolution, here defined as twice this value, is hence 1.69 ± 0.29 µA, yielding a nominal conductance resolution of 5.64 ± 0.96 µS. Conservatively, the detectability threshold was set to be slightly higher than the noise‐limited current resolution (i.e., 2 µA, instead of the nominal 1.98 µA).

The number of accessible conductance states for programming trains at 0.5 Hz is reported as a function of *V_GS,p_* in Figure 4d, revealing a trend that strongly resembles rGO‐EGTs’ transfer characteristic, with an asymmetrical behavior between the potentiation (for *V_GS,p_* > 0) and the depression (*V_GS,p_* < 0). In detail, the number of depressive accessible states is larger than the number of potentiating ones. Such asymmetry is rooted in the difference between ion dynamics and relaxation timescales, as discussed above. The depression process is slower, and the saturation of negative train pulses occurs later (i.e., at higher n) than for positive train pulses. Subsequently, for negative pulses, the difference between baselines remains for a longer time (i.e., for higher *n* values) above our resolution with respect to what happens for positive ones. As expected from a comparison between Figures 3d and 4d, small *V_GS,p_* values around *V_CNP_* identify a region where no plasticity is observed and, hence, a single conductance state is accessible.

The long‐term retention of plasticity phenomena has been assessed by applying pulse trains and recording *I_DS_* for 30 min after train removal. This measurement was performed for *V_GS,p_* = + 0.5 V (Figure 4e, top panel) and *V_GS,p_* = −0.5 V (Figure 4f, top panel), keeping the voltage fixed at *V_CNP_* after pulse train removal. The resulting currents (Figure 4e and f, bottom panels), show long‐term retention of *I_DS_* baseline modulation upon programming. This is the hallmark of LT‐potentiation (positive *V_GS,p_*, Figure 4e) and of LT‐depression (negative *V_GS,p_*, Figure 4f). LTP amplitudes, estimated as the relative percentage variations between the last *I_DS_* value and *I_DS_* before train administration, amount to roughly 30% for potentiation and 25% for depression.

The demonstrated long‐term retention of event‐related conductance modulation enables rGO‐EGTs operation as a multilevel nonvolatile memory unit, relying only on rGO‐ion interplay. Importantly, this phenomenon could in principle be exploited to achieve long‐term plasticity also with active materials different from rGO, provided that they exhibit the capability to intercalate and retain ions. In literature, similar performances are achieved only by applying large *V_GS_* (i.e., *V_GS_* > 10 V),^[^ ^44^ ^]^ by exploiting Schottky‐barrier height modulation in rGO‐junctions,^[^ ^45^ ^]^ or by developing *ad hoc* composites favoring ion intercalation.^[^ ^46^ ^]^

**Figure 5:**
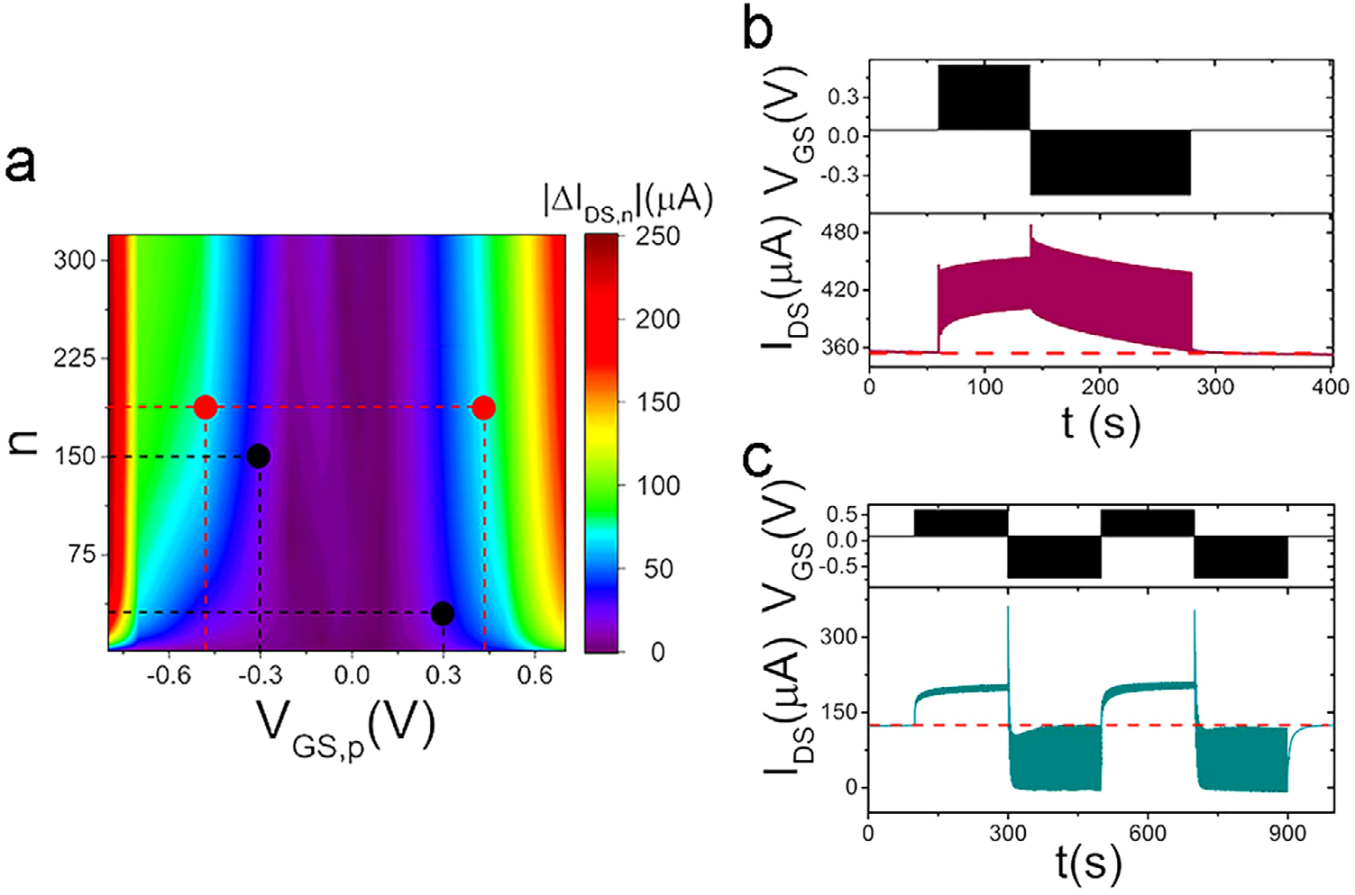
Write and erase experiments in the rGO‐EGT memory unit. a) Dependency of the LTP magnitude, |∆I_DS,n_|, upon the number of administered pulses (n) and upon V_GS,p_. b) Reversible write/erase cycling with a writing train of 40 positive pulses (V_GS,p_ = + 0.5V) followed by an erasing one of 70 negative pulses (V_GS,p_ = −0.5V). c) Reversible write/erase cycling achieved using n = 100 for both positive pulses (V_GS,p_ = + 0.5V) and negative pulses (V_GS,p_ = −0.8V).

The |*ΔI_DS,n_* | contour plot reported (**Figure** **5**a, resulting from averaged absolute values of *ΔI_DS,n_* over 3 devices), provides an operational tool‐map illustrating the magnitude of either potentiating or depressive LTP. It is a useful tool to choose and design programming pulse‐trains according to the desired final value of *I_DS_* baseline, which is a necessary prerequisite to engineer the reconfigurable multistate learning paradigms proper of neuromorphic computing.^[^ ^47^ ^]^ As an example, here is discussed the case in which perfect reversibility of memory storage – in terms of current tuning – wants to be achieved. Points of the same color in the tool‐map, referring to opposite *V_GS,p_* signs, indicate operational conditions for which potentiation and depression “cancel” each other out. This can be achieved either at symmetric *V_GS,p_*, using different *n* values for potentiation and depression (black dots) or at constant *n* by using asymmetric |*V_GS,p_* | (red dots). Figure 5b,c shows examples of reversible write/erase cycling in an individual rGO‐EGT memory unit, achieved with both strategies.

Combination of operational parameters leads to variegated tuning of LTP response upon programming cycles (Figure S11, Supporting Information). These effects constitute conceptual and technological key features toward plasticity‐driven pattern discrimination and neuromorphic computing with electrolyte‐gated organic electronic devices.

## 3 Conclusion

In this work, rGO‐EGTs were fabricated via bottom‐up electrodeposition of rGO films on gold interdigitated electrodes, eliminating the need for any surface pretreatments or GO pre‐casting and achieving fine control over thickness. Such rGO films can be engineered in rGO‐EGTs and optimized via a newly proposed cycling/reconditioning procedure, resulting in symmetric ambipolar transfer characteristics with *V_CNP_* ≈ 0 V and a good ON/OFF ratio. The proposed rGO‐EGTs show outstanding LTP upon single square pulse application and reconfigurable multistate memory upon pulse‐train administration. This is achieved directly in the working electrolyte without permanent modification of the active layer's mass, redox state, or morphology, relying solely on reversible ion intercalation. This makes rGO‐EGTs ideal candidates for applications in the wet and/or physiological environment, encompassing time‐resolved bio‐/chemo‐sensing, in situ real‐time signal processing, and higher neuromorphic functions, such as pattern discrimination and spatial co‐localization of memory and processing.

## 4 Experimental Section

### Test Patterns

Gold interdigitated electrodes were fabricated on quartz via photolithography/lift‐off (FBK, Trento, Italy). In the final layout, channel features L = 15 µm and width W = 7.5 mm (W/L = 500). As‐purchased test patterns feature a protective photoresist layer, which was removed immediately prior to use by acetone rinsing and nitrogen drying.

### Chemicals

A commercial dispersion of graphene oxide in water (0.4 wt.% in water, monolayer content >95%, 2.2 < pH < 2.5, Graphenea Inc., US) was diluted with MilliQ water to reach 0.1, 0.2, 0.3, and 0.4 wt.% used for rGO deposition and film thickness investigation. Phosphate‐buffered saline solution – PBS (1M, pH = 7.4, P3619, Sigma–Aldrich) was used as electrolyte for all the electrical and neuromorphic characterizations in Sections 2.2 and 2.3.

### rGO Electrodeposition

rGO was grown on the interdigitated electrodes starting from the GO dispersion described above, in a potentiodynamic fashion via CV in a two‐electrode configuration, using one of the two channels of a source measure unit (Keysight B2912 A). Short‐circuited interdigitated source and drain electrodes were connected to the “high‐force” terminal, while a Pt‐wire was connected to the grounded “low‐force” terminal. The liquid GO dispersion contacts both the short‐circuited interdigitated electrodes and the Pt‐wire, closing the circuit and allowing electrochemical reactivity. A strictly reductive CV was obtained by scanning the voltage of the high‐force terminal from −1.3V to V_end_ (where V_end_ varies from −2.3 to −2.8 V with an interval of −0.1 V), with a scan rate of 90 mV s^−1^. To assess the dependency of the thickness of electrodeposited‐rGO films on the concentration of GO dispersion, CV was performed with a fixed V_end_ of −2.8 V using the GO dispersions described above.

### Atomic Force Microscopy

The surface topography of the electrodeposited rGO on FBK quartz substrate was investigated using a Park XE7 AFM System (Park Systems, Suwon, Korea), operating in tapping mode, in air, at room temperature. The instrumentation features pre‐mounted silicon cantilevers (OMCL‐AC160TS, Olympus Micro Cantilevers, Tokyo, Japan) with aluminum backside reflective coatings, tip curvature radius of ≈7 nm, elastic constant of ≈26 N/m, and resonance frequency of ≈300 Hz. The thickness of each electrodeposited‐rGO film was calculated as the average of nine different regions across the scratch edge. The roughness value was calculated from 5 µm × 5 µm topographic images of the surface acquired for three different samples. Data were presented as mean ± SEM.

### Electrical Characterization

*I–V* characteristics were carried out in PBS as working electrolyte, using a two‐channel Source/Measure Unit (Keysight, B2912A). The devices were operated in a common‐source/common‐ground configuration, connecting the source electrode to the grounded “low” terminals of the two channels, whereas gate and drain electrodes were connected to the “high” terminals of channel 1 and channel 2, respectively. Transfer characteristics during cycling were acquired sweeping V_GS_ from −0.4 to 1.5 V (scan rate = 90 mV/s), while keeping fixed V_DS_ equal to + 0.3 V. After the reconditioning step, transfer characteristics were acquired sweeping V_GS_ from −0.8 to 1.2 V (scan rate = 90 mV/s) while V_DS_ was kept fixed at + 0.3 V. Furthermore, rGO‐EGT 3D‐characteristics (Figure 2f) were collected sweeping V_GS_ from −0.8 to + 1.2 V, changing the V_DS_ value between the V_GS_ sweep cycles from −0.3 to 0.3 V with voltage steps of 0.15V.

Neuromorphic response was investigated with the already described operational layout, providing single square voltage pulses and square pulse trains at the presynaptic terminal (i.e., gate electrode) by means of custom‐designed software, while acquiring at the postsynaptic terminal (i.e., rGO channel) the postsynaptic current, I_DS_. The administered pulses were designed with their baseline set to the V_CNP_ of devices, varying their amplitude (V_GS,p_) between −0.8 to + 0.7 V in both single pulses and square trains, while keeping their time duration (Δt) fixed at 1s (duty cycle of ½).

### Data Analysis

All the data presented herein were analyzed using Matlab (version 9.10, Mathworks, Natick, MA, USA) and plotted using OriginPro2016. Figure panels were assembled in Adobe Photoshop CS6, and 3D‐device schematics were sketched in SketchUp Make 2017. AFM images were analyzed using Park Systems XEI software (Park Systems, Suwon, Korea) and Gwyddion freeware (version 2.62 http://gwyddion.net/) in order to extract the root‐mean‐square roughness and the thickness of the rGO films.

## Conflict of Interest

The authors declare no conflict of interest.

## Supporting information



Supporting Information

## Data Availability

The data that support the findings of this study are available from the corresponding author upon reasonable request.
