# Supplementary material for: Electrodeposited Reduced Graphene Oxide Enables Long‐Term Memory in Neuromorphic Ambipolar Electrolyte‐Gated Transistors
Source: Small. 2025 May 16;21(27):2502768. doi: 10.1002/smll.202502768 (PMC12243726; doi:10.1002/smll.202502768)
Supplement: Supplementary file 1 — Supporting Information [file SMLL-21-2502768-s001.docx]

Supplementary Information for:

**Electrodeposited Reduced Graphene Oxide Enables Long-Term Memory in Neuromorphic Ambipolar Electrolyte-Gated Transistors**

*Maryam Abouali^1†^, Federico Rondelli^1^,^2†^, Matteo Genitoni^1,2^, Mauro Murgia^1,3^, Michele Di Lauro^1^*, Luciano Fadiga^1,2^, and Fabio Biscarini^1,4^*

This work is dedicated to the beloved memory of Prof. Paolo Biscarini.

M. Abouali, F. Rondelli, M. Genitoni, M. Murgia, L. Fadiga, F. Biscarini

Center for Translational Neurophysiology of Speech and Communication, Fondazione Istituto Italiano di Tecnologia (IIT-CTNSC), via Fossato di Mortara 17/19, Ferrara 44121, Italy

F. Rondelli, M. Genitoni, L. Fadiga

Sezione di Fisiologia, Dipartimento di Neuroscienze e Riabilitazione, Università di Ferrara, via Fossato di Mortara 17/19, Ferrara 44121, Italy

M. Murgia

Istituto per lo Studio dei Materiali Nanostrutturati (CNR-ISMN), National Research Council, via Gobetti 101, Bologna 40129, Italy

M. Di Lauro

Center for Translational Neurophysiology of Speech and Communication, Fondazione Istituto Italiano di Tecnologia (IIT-CTNSC), via Fossato di Mortara 17/19, Ferrara 44121, Italy

E-mail: [michele.dilauro@iit.it](mailto:michele.dilauro@iit.it)

F. Biscarini

Dipartimento di Scienze della Vita, Università di Modena e Reggio Emilia, Via Campi 103, Modena 41125, Italy

†These authors contributed equally to this work.

**Content List:**

- Figure S1. Thickness assessment;
- Figure S2. Dependency of electrical performance of rGO-EGTs on GO dispersion concentration;
- Table S1. Figures of merit related to 30th transfer curves of rGO-EGT fabricated with different concentrations of GO dispersion;
- Figure S3. Dependency of exchanged charge and *Vend* during electrodeposition;
- Figure S4. Transconductance profiles comparison upon reconditioning;
- Figure S5. Device figures of merit
- Figure S6. Effect of rGO-EGTs fabrication steps on the morphology of rGO channels;
- Figure S7. Intra-pulse current fitting in single pulse rGO-EGT neuromorphic response;
- Figure S8. Dependency of single-pulse responses of the as-fabricated rGO-EGT devices on the number of transfer cycles and on the reconditioning step;
- Figure S9. 2D representation of dependency of train pulse responses of the rGO-EGT devices on the number of pulses and voltage amplitudes;
- Figure S10. The dependency of train pulse responses of the rGO-EGT devices on the number of transfer cycles and the reconditioning step;
- Figure S11. Multi-level memory operation.


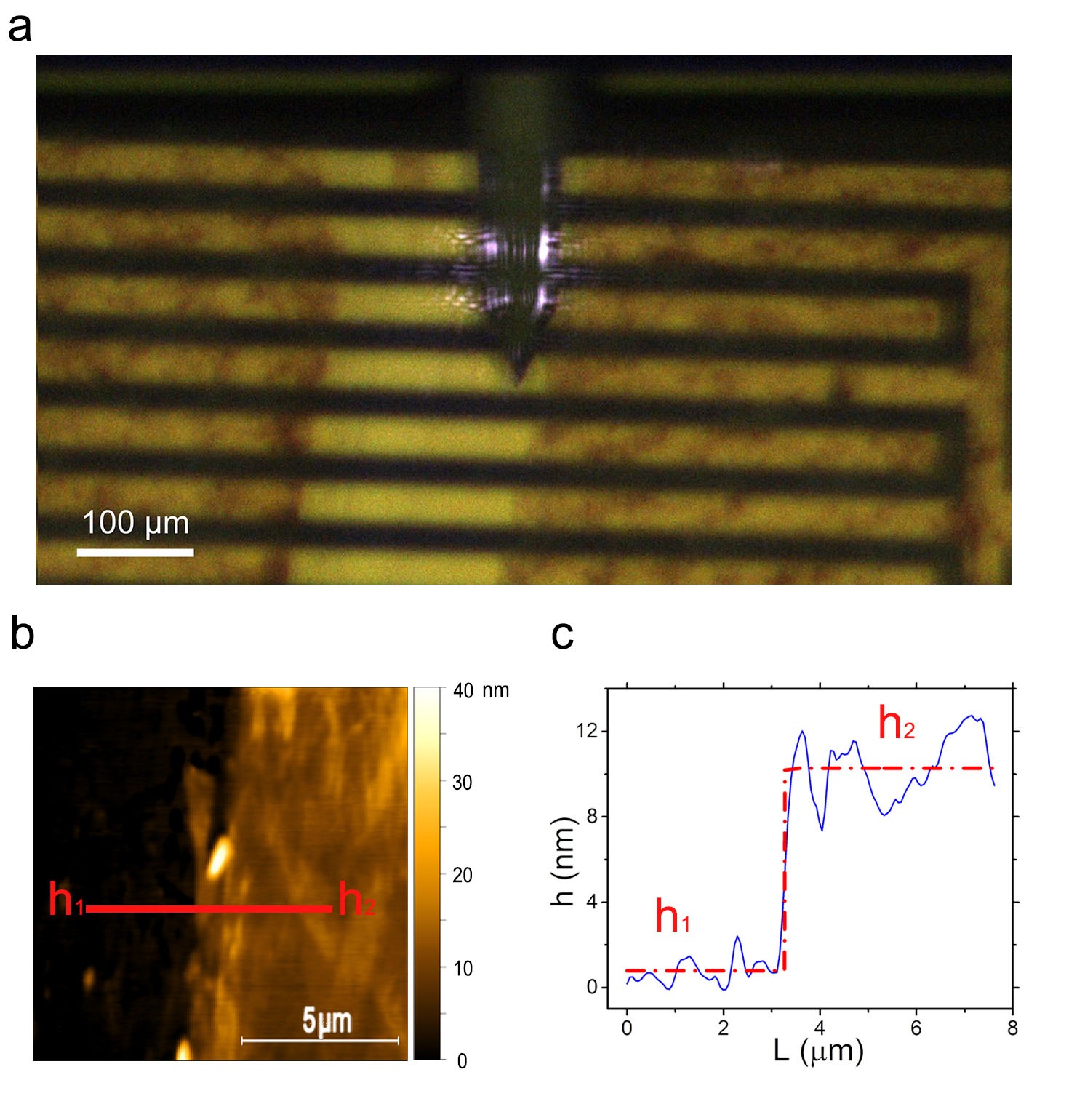


**Figure S1.** **Thickness assessment**. **a,** Scratched zone on the rGO-EGT channel, used for thickness measurements. **b**, AFM image of the edge of the electrodeposited-rGO thin film and, **c**) corresponding height (h) profile of length (L) across the scratched edge. Dashed line is step fitting of the height profile, with the usual form h=h_1_ for L<L_0_ and h=h_2_ for L>L_0_, with L_0_ as the scratch boundary, used to extract thickness as h_2_-h_1_.


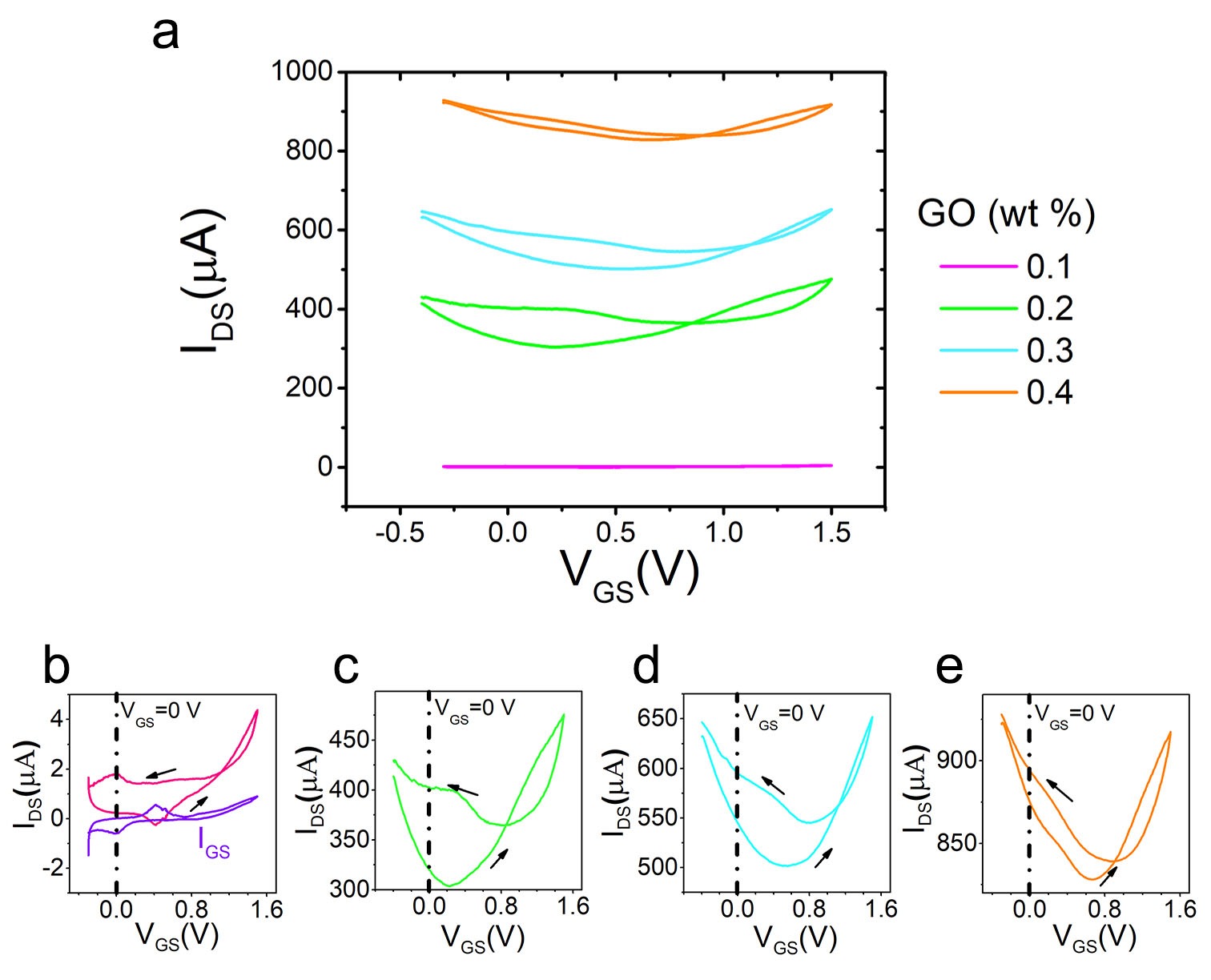


**Figure S2.** **Dependency of electrical performance of rGO-EGTs** **on GO dispersion concentration.** **a**, *I_DS_* vs *V_GS_* plots related to the last conditioning step (*i.e.*, the 30-th transfer scan) for rGO-EGT devices fabricated starting from GO dispersion at varying concentrations. **b-d**, Rescaling of curves for more details, for devices based on (**b**) 0.1 wt% GO dispersion (with details of *I_GS_* to show the absence of ambipolarity and the scan-induced capacitive contribution as main current source in the p-branch), (**c**) 0.2 wt% GO dispersion, (**d**) 0.3 wt% GO dispersion, and (**e**) 0.4 wt% GO dispersion.

**Table S1.** **Figures of merit related to 30^th^ transfer curves of rGO-EGT fabricated with different concentrations of GO dispersion.**

| Concentration of GO dispersion (wt %) | *V_CNP_* (V) | ON/OFF ratio-n branch | ON/OFF ratio-p branch |
| --- | --- | --- | --- |
| 0.1 | N/A | N/A | N/A |
| 0.2 | 0.22 | 1.36 | 1.56 |
| 0.3 | 0.55 | 1.26 | 1.29 |
| 0.4 | 0.66 | 1.11 | 1.10 |

* N/A: not available, since – in rGO-EGT based on 0.1 wt% GO dispersions – the current component related to ionic displacement is comparable with the current elicited in the channel and, hence, figures of merit are not unambiguously attributable to the channel.





**Figure S3.** **Dependency of exchanged charge and *V_end_* during electrodeposition**. The charge, Q, is estimated by integration of the cyclic voltammetry current of partial electrodepositions over time. The exponential fit here is intended as a generic guide for the eye.


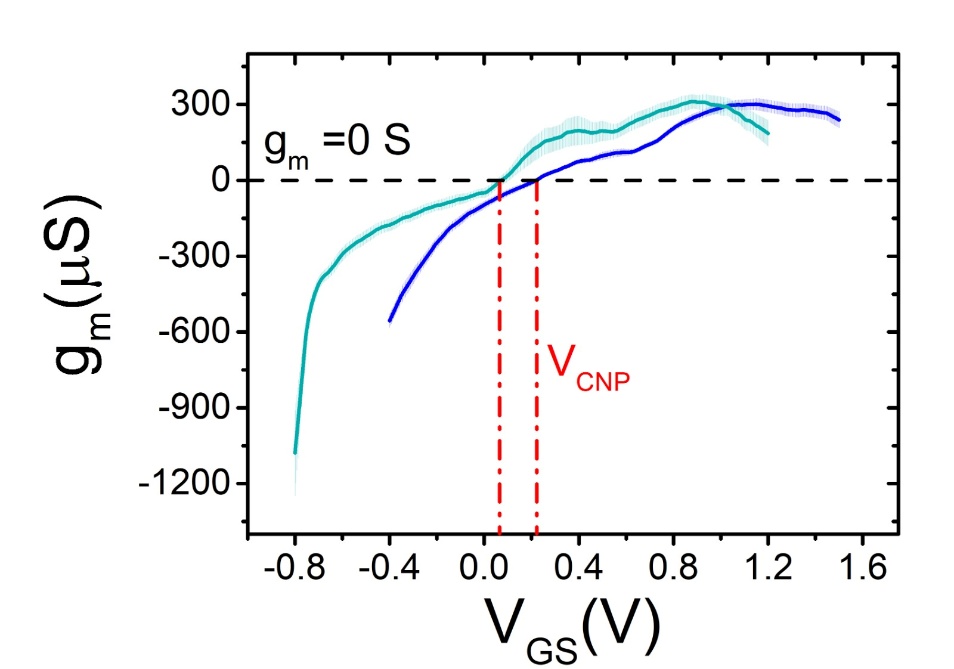


**Figure S4. Transconductance profiles comparison upon reconditioning.** Improved transconductance (*g_m_*) in rGO-EGTs after the reconditioning step (emerald green solid line, n_sample_ = 8, standard error of the mean as error bars) compared to the *g_m_* profile of rGO-EGTs after the 30^th^ *V_GS_* sweep (blue solid line n_sample_ = 8, standard error of the mean as error bars). Negative shift of *V_CNP_* after reconditioning is shown as the point at null *g_m_* (red dot-dashed lines).


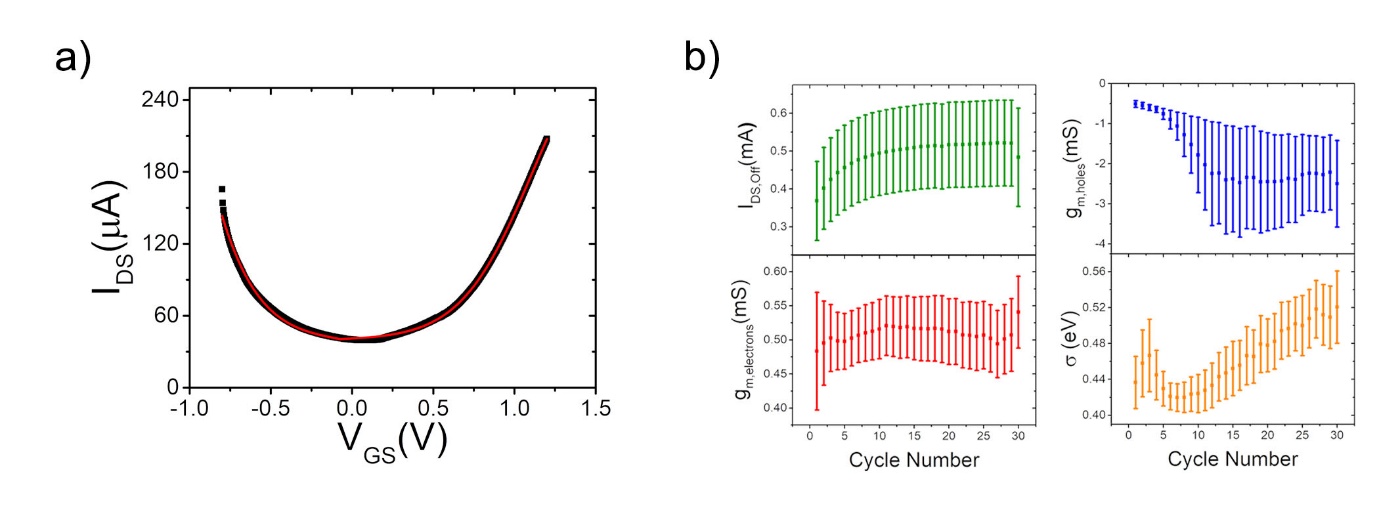


**Figure S5. Device figures of merit.** a) Typical forward scan transfer characteristic (black squares) with superimposed fitting with the model from ref. 40 (red solid line), showing excellent agreement between experimental data and analytical model; b) time evolution of the device figures of merit derived from the analytical model during the conditioning procedure. Notice how OFF current increases up to a plateau, p-type transconductance decreases and n-type transconductance remains constant. Importantly, the disorder parameter σ shows a marked increase, hinting at film rearrangement upon wetting and ion intercalation/de-intercalation. The averaged parameters of the final device layout, after re-conditioning, are I_DS,off_ = 180 ± 50 μA, g_m,electrons_ = 0.68 ± 0.04 mS, g_m,holes_ = 1.49 ± 0.40 mS, α = 0.104 ± 0.036, σ = 0.517 ± 0.037 eV, V_CNP_= -0.040 ± 0.035 V and ε = 0.47 ± 0.07 eV.


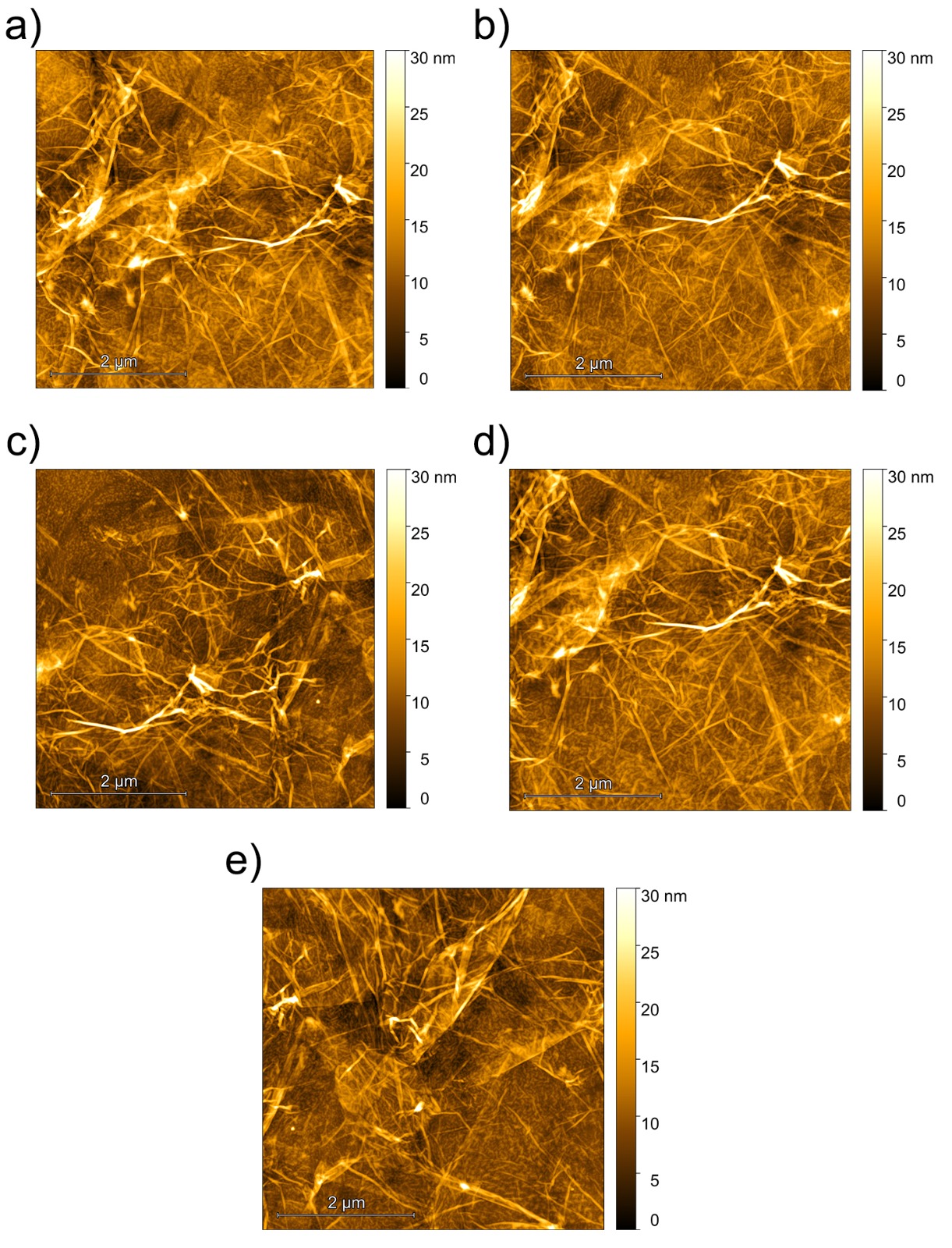


**Figure S6.** **Effect of rGO-EGTs fabrication steps on the morphology of rGO channels**. **a-e,** AFM images collected from an rGO channel: (**a)** after electrodeposition, (**b)** after 5 transfer cycles, (**c)** after 15 transfer cycles, (**d)** after 30 transfer cycles, (**e)** after reconditioning.


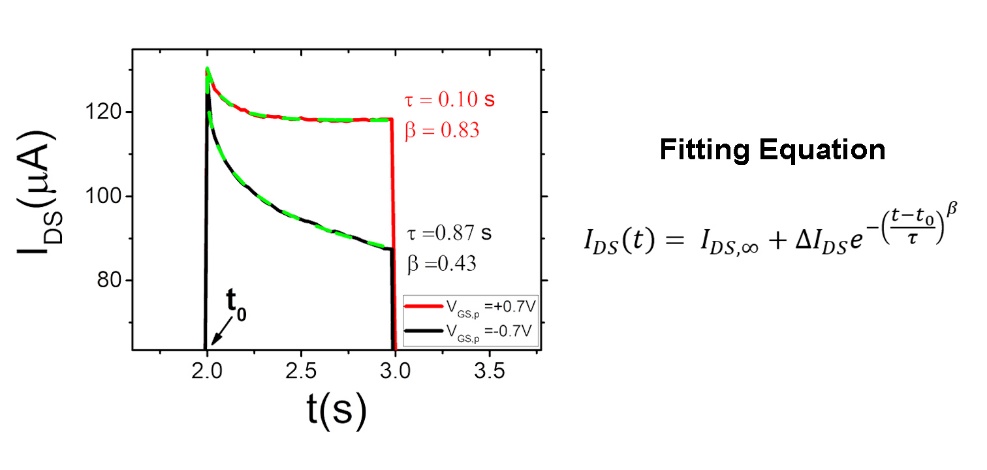


**Figure S7.** **Intra-pulse current fitting in single pulse rGO-EGT neuromorphic response.** Stretched exponential fitting (green dashed lines) of *I_DS_* within the positive and negative pulses (solid lines).


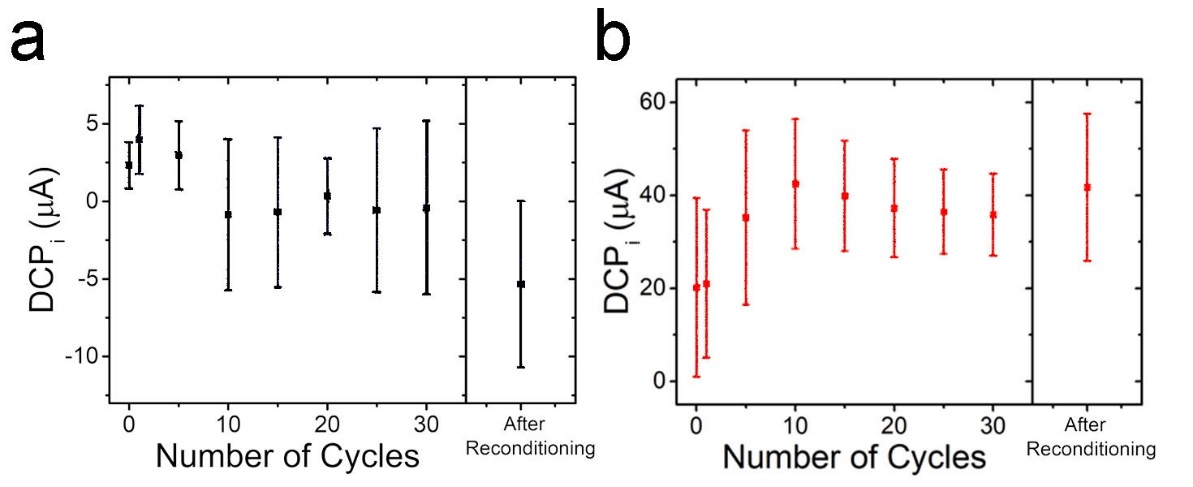


**Figure S8.** **Dependency of single-pulse responses of the as-fabricated rGO-EGT devices on the number of transfer cycles and on the reconditioning step.** **a**, Response of the device to -0.7 V pulse amplitude. **b**, Response of the device to +0.7 V pulse amplitude. (n_sampl_**_e_**=3, standard error of the mean as error bars).


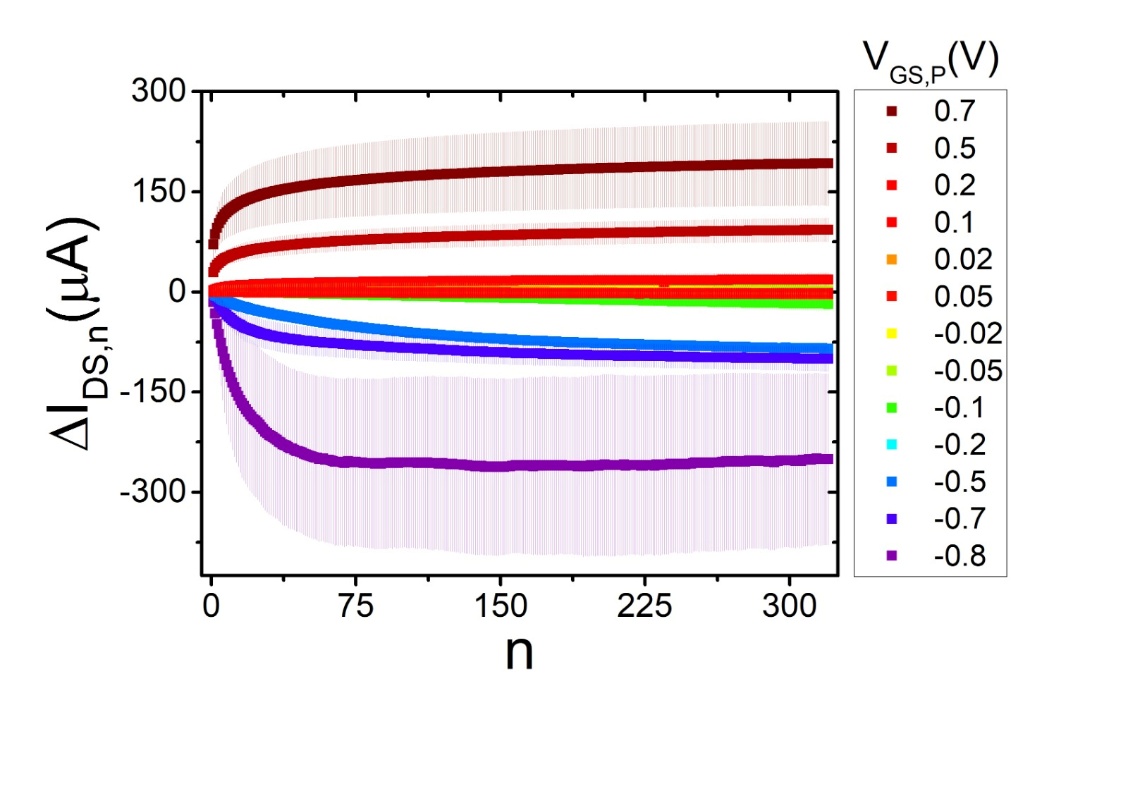


**Figure S9.** **2D representation of dependency of train pulse responses of the rGO-EGT devices on the number of pulses and voltage amplitudes.**


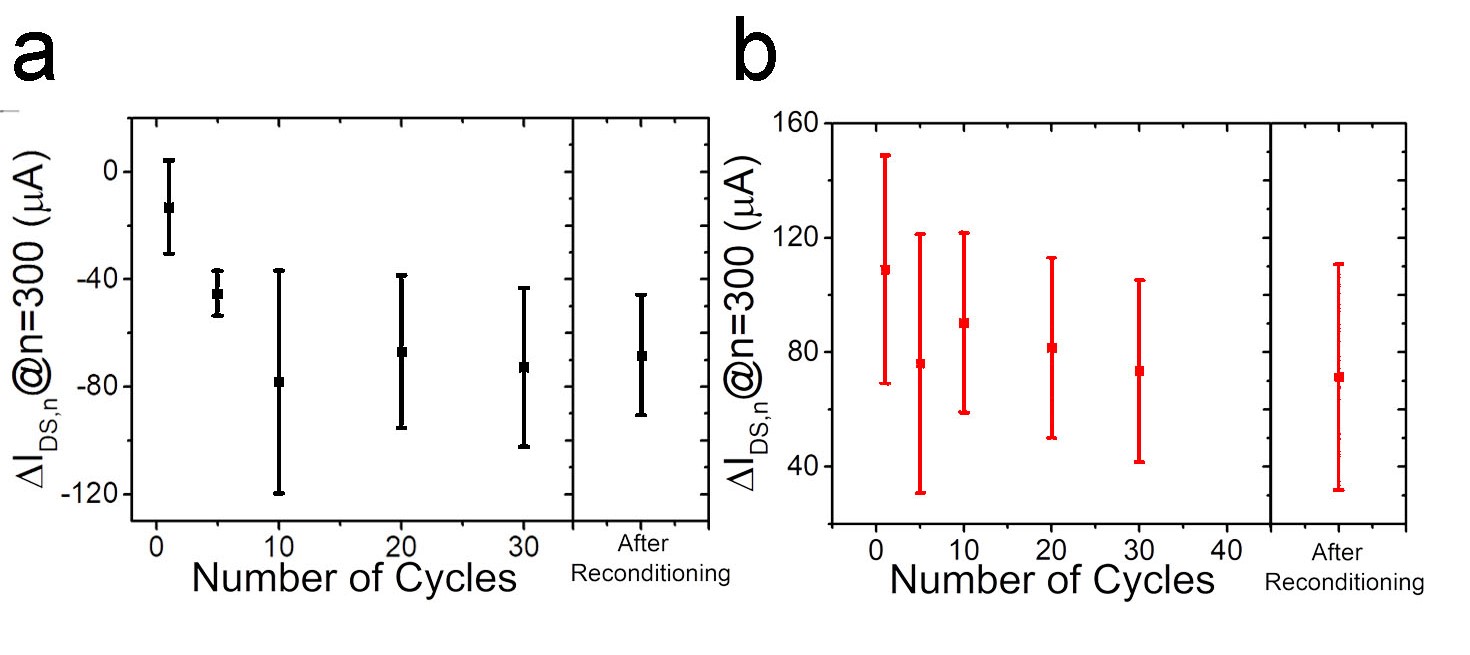


**Figure S10.** **The dependency of train pulse responses of the rGO-EGT devices on the number of transfer cycles and the reconditioning step.** **a**, Response of the device to train pulses with -0.7 V amplitude. **b**, Response of the device to train pulses with +0.7 V amplitude. (n_sample_= 3, standard error of the mean as error bars).


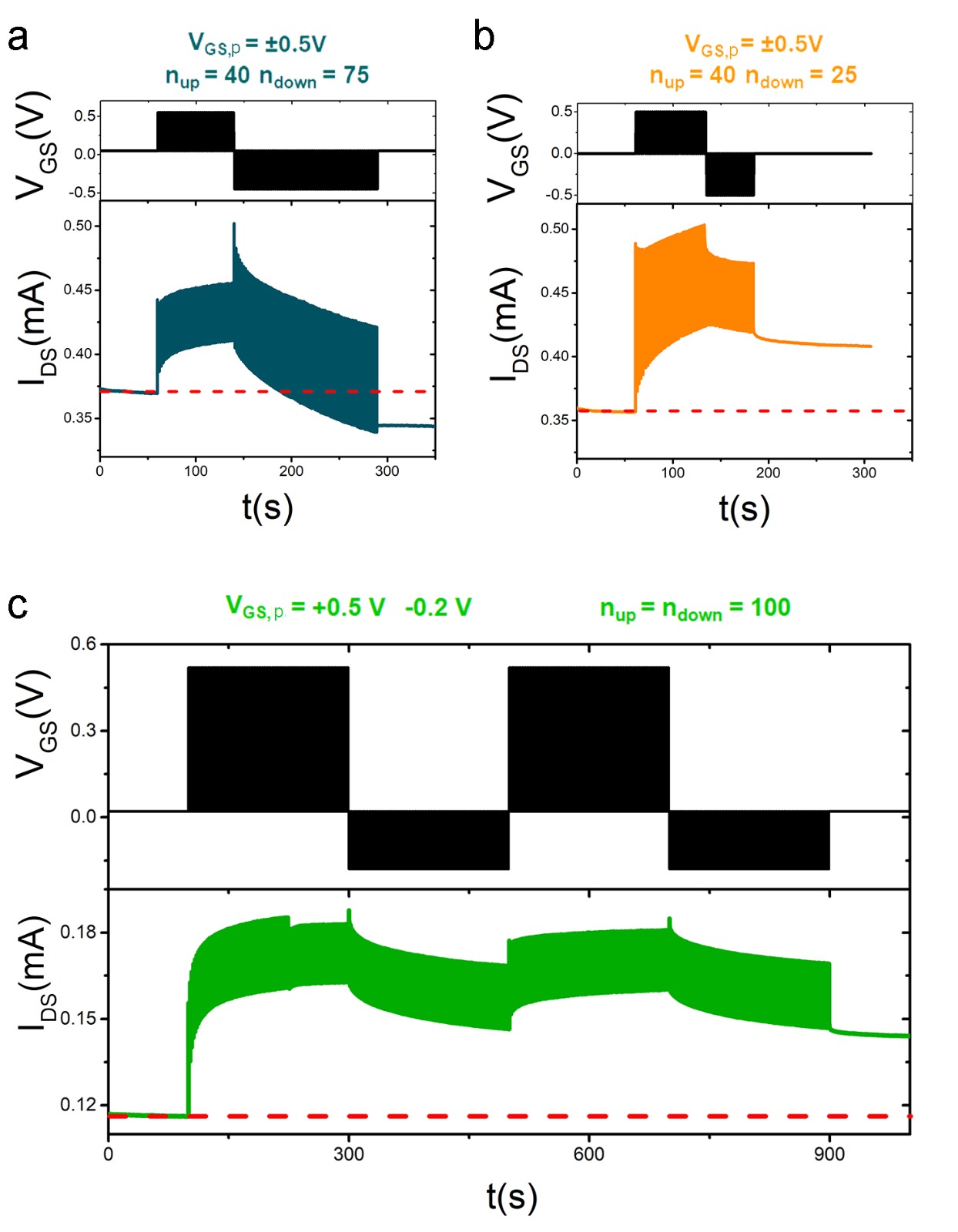


**Figure S11.** **Multi-level memory operation.** The performance of the rGO-EGTs as non-volatile multi-state memory indicating their ability to be programmed at will, according to the tool-map from Fig. 5a. **a-b**, Voltage pulsing with same |*V_GS,p_|* and fixed number of positive pulses (*n_up_*), while changing the number of negative pulses (*n_down_*) resulting in depressive (**a**) or facilitative (**b**) retention. **c**, Voltage pulsing with same *n* for both positive and negative trains, while changing *V_GS,p_* from positive pulsing to negative pulsing.
